# *Fusarium graminearum* and Its Interactions with Cereal Heads: Studies in the Proteomics Era

**DOI:** 10.3389/fpls.2013.00037

**Published:** 2013-02-28

**Authors:** Fen Yang, Susanne Jacobsen, Hans J. L. Jørgensen, David B. Collinge, Birte Svensson, Christine Finnie

**Affiliations:** ^1^Enzyme and Protein Chemistry, Department of Systems Biology, Technical University of DenmarkLyngby, Denmark; ^2^Department of Plant and Environmental Sciences, Faculty of Science, University of CopenhagenFrederiksberg C, Denmark

**Keywords:** *Fusarium graminearum*, *Fusarium* head blight, pathogenicity, plant defense response, proteomics

## Abstract

The ascomycete fungal pathogen *Fusarium graminearum* (teleomorph stage: *Gibberella zeae*) is the causal agent of *Fusarium* head blight in wheat and barley. This disease leads to significant losses of crop yield, and especially quality through the contamination by diverse fungal mycotoxins, which constitute a significant threat to the health of humans and animals. In recent years, high-throughput proteomics, aiming at identifying a broad spectrum of proteins with a potential role in the pathogenicity and host resistance, has become a very useful tool in plant-fungus interaction research. In this review, we describe the progress in proteomics applications toward a better understanding of *F. graminearum* pathogenesis, virulence, and host defense mechanisms. The contribution of proteomics to the development of crop protection strategies against this pathogen is also discussed briefly.

## Introduction

The pathogen *Fusarium graminearum* causes devastating head blight of small grain cereals including wheat and barley. *Fusarium* head blight (FHB), as a global problem, has great economic impact on the cereal industry due to the reduced grain yield and quality as well as to the contamination by diverse mycotoxins, including deoxynivalenol (DON) and zearalenone, which are harmful for humans and animals.

**Figure 1:**
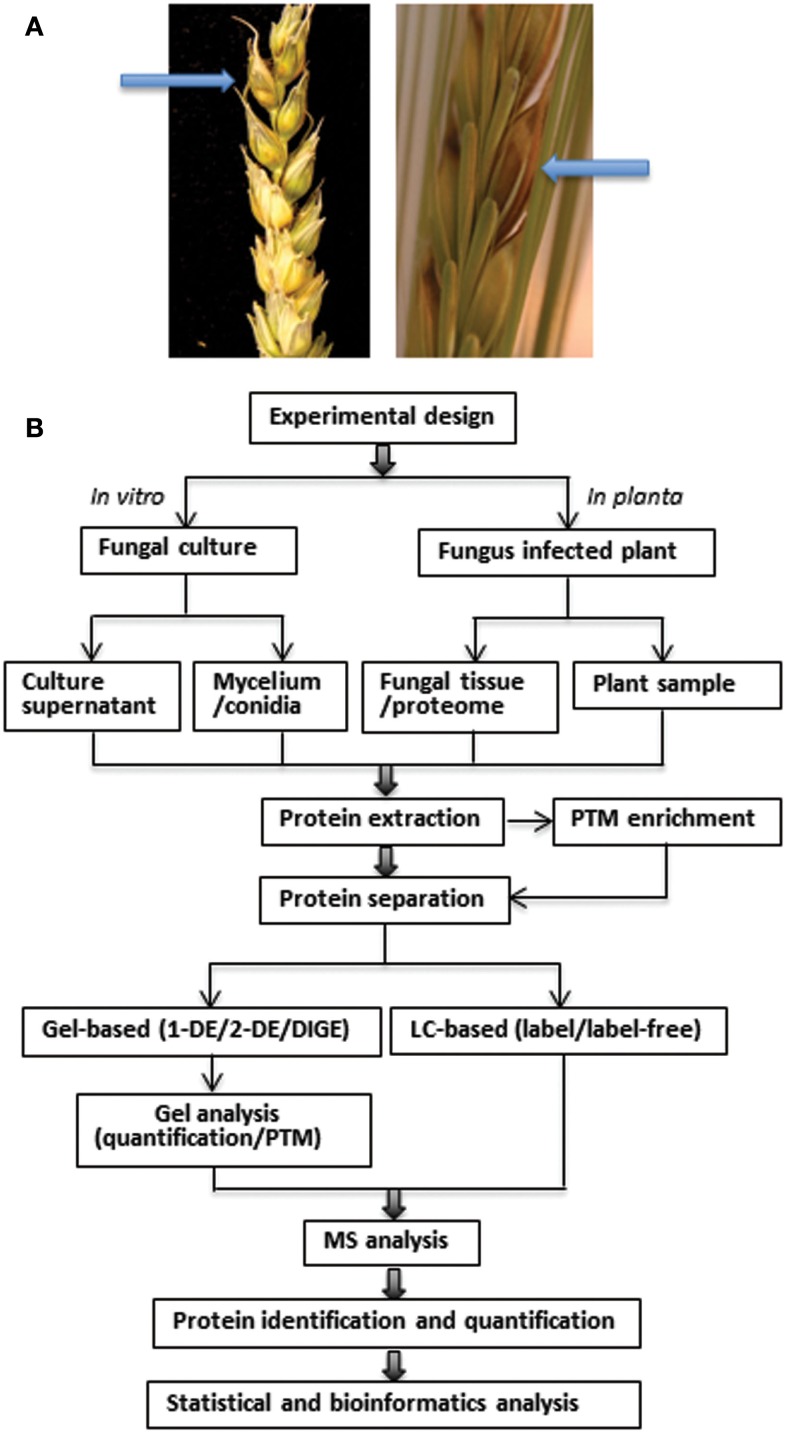
**(A)** Symptoms (indicated by arrows) of *Fusarium* head blight in the spikelets of wheat (left) and barley (right). Bleaching and dark necrotic lesions can be observed in the infected wheat spikelets. Infected barley spikelets show a browning or water-soaked appearance. The spikelets were point-inoculated with *F. graminearum* at anthesis and photographed at 6 dai by Jens Due Jensen and David B. Collinge, University of Copenhagen. **(B)** Schematic overview of proteomics workflow in phytopathogenic fungi. The major steps include experiment design, sampling, protein extraction, PTM enrichment, protein separation, MS analysis, protein identification, and quantification, followed by bioinformatics analysis of the data.

The disease (Figure 1A) is initiated by deposition of spores on or inside flowering spikelets (Bushnell et al., 2003). Fungal hyphae develop on the exterior surfaces of florets and glumes, rather than by direct penetration through the epidermis, prior to the colonization of anthers, stigmas, and lodicules (Bushnell et al., 2003). The fungus spreads in wheat from spikelet to spikelet through the vascular tissue in the rachis and rachilla (Trail, 2009) and this is associated with the production of DON, a virulence factor (effector molecule) causing tissue necrosis (Jansen et al., 2005). In barley, spread of the disease is limited and virulence does not appear to be due to the presence of the toxin (Maier et al., 2006). The fungus apparently exhibits a brief biotrophic phase before switching to the necrotrophic phase, when vigor of colonization increases by the fungus and eventually the plant cells die (Trail, 2009).

As a result of its devastating effects, *F. graminearum* has been under intense investigation for many years to understand the genetic basis of the life cycle, pathogenicity, evolution, and population biology. Availability of the full genome sequence (Ma et al., 2010) considerably revitalizes research of gene function in *F. graminearum*. In addition to classical biochemical, genetic, molecular biological, and plant pathology approaches, several “omics” techniques are employed in the studies of *F. graminearum* and its interactions with hosts. Transcriptome and metabolome analysis have been conducted in *F. graminearum* during the invasion of hosts, sexual development, and conidial germination, in response to azole fungicide and/or in *F. graminearum* mutants as well as in barley and wheat during infection to understand defense responses (reviewed by Kazan et al., 2012). *In silico* prediction of the secretome of *F. graminearum* has also been performed to identify potential pathogenicity factors and effectors (Brown et al., 2012). Proteomics, as the core technology in functional genomics, allows interpretation of gene function, determination of protein abundance, interactions, modifications, locations, and implications in development and environmental responses (Wright et al., 2012). In the present review, we focus on the recent progress made by using proteomics techniques to enhance the understanding of cellular and molecular mechanisms of *F. graminearum* pathogenicity and virulence as well as the host defense responses.

## Proteomics Techniques in Phytopathogenic Fungi

Proteome analysis of phytopathogenic fungi and their interactions with hosts has increased dramatically over the last years, because of the technical development of “omics” and bioinformatic tools, and the growing number of fungal genomes being sequenced. Investigations in this area mainly are (i) identification of mycelial, conidial, and secreted proteins across a range of fungal species by establishing reference proteome maps of these fungal structures. Proteome profiles are conducted and/or compared between species, races, mutants, growth, development stages, and growth conditions (Gonzalez-Fernandez et al., 2010), in particular during spore germination, hyphal penetration, appressorium formation, toxin production, and secretion (vanKan, 2006), and (ii) plant-fungus interactions to study infection cycles, to identify pathogenicity factors and to study plant defense responses. Analysis of proteins of some fungal species *in planta* is limited due to the fact that it is difficult to isolate fungal tissues from the infected hosts and that the fungal biomass constitutes a small portion of the total biological material resulting in the dominance of plant proteins. Besides fungi with agricultural interest, such as *Botrytis cinerea*, *Sclerotinia sclerotiorum*, and *F. graminearum* (reviewed by Gonzalez-Fernandez and Jorrin-Novo, 2012), important studies employing a diversity of proteomics techniques have been performed on major crops, including rice, maize, wheat, and barley interacting with fungal pathogens, in addition to *Arabidopsis thaliana* (reviewed by Gonzalez-Fernandez et al., 2010).

The workflow of proteomic analysis in phytopathogenic fungi is shown in Figure 1B. Experimental design and sampling reflect the aim of the study, i.e., whether it has focus on the fungus or the plant under the chosen conditions. The protein extraction protocol is a very critical step and determines which proteins are available for analysis. This step is particularly challenging for both plants and fungi, because of their robust cell walls in addition to proteases and different non-protein components which can interfere both with the population and quality of the proteins and their subsequent separation (Hurkman and Tanaka, 2007). Post-translational modifications (PTMs) can also be analyzed using proteomics, but require selective enrichment and purification strategies due to their reversible and labile nature and low stoichiometric abundances. Some PTMs, such as phosphorylation, glycosylation, acetylation, phenylation, *S*-nitrosylation, and ubiquitylation, are involved in signal transduction during plant-microbe interactions and have been analyzed by proteomics (Jayaraman et al., 2012).

Protein separation in the majority of earlier proteomics studies was based on two-dimensional gel electrophoresis (2-DE) coupled with conventional staining methods. Difference gel electrophoresis (DIGE), where samples are labeled differentially with fluorophores, allows distinction between proteins obtained in different samples that can be resolved on the same gel. This can address the issues of both sensitivity and gel variability in 2-DE (Wright et al., 2012). However, DIGE suffers from the same problems as traditional 2-DE, especially in relation to the resolution of hydrophobic proteins and proteins exhibiting extreme pIs and molecular weights. Currently, gel-free techniques for separating peptides become standard for large-scale shotgun proteomics, which can overcome some of the limitations of the gel-based approach. The methods are based on the pre-fractionation of peptide mixtures by mono-dimensional LC or multidimensional protein identification technology (MudPIT) such as strong cation exchange (SCX) combined with reversed phase chromatography (Gilmore and Washburn, 2010).

Mass spectrometry (MS), consisting of an ion source, a mass analyzer, and a detector, is the most common technique for unbiased protein identification (Aebersold and Mann, 2003). The various techniques for ionizing samples include matrix assisted laser desorption/ionization (MALDI) and electrospray ionization (ESI). The mass analyzers include time-of-flight (TOF), ion trap, quadruple, orbitrap, and fourier transform ion cyclotron resonance. In MS/MS, specific precursor ions produced in the initial mass analyzer are chosen and fragmented, resulting in sequence-informative fragment ion spectra. Fragmentation methods can be collision-based (e.g., CAD and HCD) or electron-based (e.g., ECD and ETD) dissociation (Coon, 2009). Observed ion spectra are compared against databases containing known protein sequences by search algorithms (e.g., SEQUEST, Mascot, and OMSSA) for protein identification.

Comparative proteomics can be based on the traditional pre-staining of 2-DE gels such as Coomassie Blue staining, silver staining, and fluorescence staining and the modern label-free or labeling approaches at the MS stage, followed by the statistical, and bioinformatics analysis to determine the significance of data. Isotope-assisted quantification methods include *in vitro* chemical (e.g., ICAT, iTRAQ, TMT, and ^18^O) and *in vivo* metabolic (e.g., SILAC and ^15^N-labeling) labeling of biological samples. In chemical labeling, distinct protein samples are labeled with heavy and light isotopes or isobaric tags, pooled, and compared by MS. Stable isotope labeling of amino acids in cell culture (SILAC) or plants that are grown on media supplemented with heavy isotope-containing amino acids, allows for labeling of proteins as they are synthesized (Ong et al., 2002). The relative ratio of protein from different samples is determined by the ratios of signal intensities of the labeled peptides that are common to the samples in MS analysis. Label-free quantification compares samples based on the measurement of changes in peptide peak areas or peak heights in chromatography and peptide peak intensity in MS or the spectral counting of identified proteins after MS/MS analysis (Neilson et al., 2011).

## *Fusarium graminearum* Proteome Analyses

Proteomics studies conducted on *F. graminearum* have focused mainly on the secretome and impact of DON (Table 1). This is due to the important roles of secreted proteins and DON in pathogenicity. The first *in vitro* gel-based secretome study in *F. graminearum* was performed in a culture with a medium containing either glucose or hop cell walls. Here, 23 and 84 unique proteins were identified, respectively, mainly involved in cell wall polysaccharide degradation (Phalip et al., 2005). Using LC-MS/MS, 229 fungal proteins, mostly glycoside hydrolases and proteases, were identified in the secretome of *F. graminearum* during growth on 13 synthetic media (Paper et al., 2007). To closely mimic the nutritional situation of the fungus *in planta*, Yang et al. (2012) employed a gel-based proteomics approach to access the secretome in the growth cultures with barley or wheat flour as the sole nutrient source, resulting in the identification of 69 unique fungal proteins including enzymes involved in the degradation of cell walls, starch, and proteins. Secreted proteins differing in accumulation between wheat and barley flour media were mainly involved in fungal cell wall remodeling and the degradation of plant cell walls, starch, and proteins. To analyze the effect of DON production in host infection process, *F. graminearum* was grown on a medium promoting trichothecene biosynthesis (Taylor et al., 2008). Here, comparative proteomics showed 130 differentially expressed fungal proteins, of which proteins potentially involved in virulence were up-regulated, whereas down-regulated proteins were primary metabolic enzymes, chaperones, and proteins involved in translation.

**Table 1:** **Original proteomics papers published on *F. graminearum* and its interactions with wheat and barley**.

Growth conditions	Sampling times	Sample materials	Proteomics techniques	Remarks	Reference
In the growth media containing either glucose or hop cell walls	6 d, 9 d	Culture supernatants	1-DE, 2-DE, LC-MS/MS	Analysis of the fungal *in vitro* secretomes	Phalip et al. (2005)
In the synthetic media containing polysaccharide supplements	7 d	Culture supernatants	1-DE, LC-MS/MS	High-throughput analysis of the fungal *in vitro* secretomes	Paper et al. (2007)
In the wheat grains	Maturity	Fungal secretome	1-DE, LC-MS/MS	Analysis of the fungal *in planta* secretomes	Paper et al. (2007)
In the growth medium promoting trichothecene biosynthesis after 2-day growth in the rich medium	0, 4 d, 9 d, 12 d	Fungal tissues	iTRAQ, LC-MS/MS 2-DE, MS/MS	*In vitro* time course study of the changes in fungal intercellular proteomes due to the induction of trichothecene production	Taylor et al. (2008)
In the growth media containing only barley or wheat flour	7 d	Culture supernatants	2-DE, MALDI-MS/MS	Study of the fungal *in vitro* secretomes under growth conditions which mimic *in planta* nutritional situation	Yang et al. (2012)
In the growth medium with limited nitrogen after 2-day growth in the rich medium	0, 6 h, 12 h	Fungal tissues	2-DE, MALDI-MS, 1-DE, IMAC, TiO_2_, LC-MS, SAX, IMAC, LC-MS/MS	Analysis of the fungal phosphoproteomes under the *in vitro* growth condition that activates trichothecene pathway	Rampitsch et al. (2010)
In the growth medium with unlimited nutrients	1 d	Fungal tissues	SCX, IMAC, LC-MS/MS	Analysis of the fungal *in vitro* phosphoproteomes	Rampitsch et al. (2012)
Virus-free and -infected strains grown in the complete medium	5 d	Fungal tissues	2-DE, LC-MS/MS	Study of the fungal proteomes in response to viral infection	Kwon et al. (2009)
In the resistant wheat spikes	6 h, 12 h, 24 h	Wheat spikes	2-DE, MALDI MS	Study of the differential expressed wheat proteins in response to fungal infection	Wang et al. (2005)
In the susceptible and resistant wheat spikes	5 d	Wheat spikelets	2-DE, LC-MS/MS	Study of the differential expressed wheat proteins in response to fungal infection	Zhou et al. (2005)
In the susceptible wheat spikes	1 d, 2 d, 3 d	Wheat spikelets	2-DE, LC-MS/MS	Identification of wheat proteins regulated by the fungus and fungal expressed proteins *in planta*	Zhou et al. (2006)
In the susceptible wheat ears	5 d, 15 d, 25 d	Wheat ears	DIGE, MALDI MS/MS	Investigation of the changes in xylanase inhibitors (iso)forms of wheat due to fungal *Tir5* mutant infection	Dornez et al. (2010)
In the susceptible and resistant wheat spikes	12 h	Wheat spikes	2-DE, MALDI MS	Study of the differential expressed wheat proteins and genes in response to fungal infection	Ding et al. (2011)
In the moderate resistant wheat spikes	48 h	Wheat spikes	2-DE, MALDI MS	Study of the differential expressed wheat proteins in response to fungal infection	Shin et al. (2011)
In wheat carrying either resistant or susceptible alleles at the *Fhb 1* locus	72 h	Wheat spikelets	LC-MS/MS, spectral counting	Identification of mechanisms of resistance governed by the FHB resistance locus *Fhb 1*	Gunnaiah et al. (2012)
In emmer heads and co-colonization with *Fusarium culmorum*	Maturity	Emmer grains	2-DE, LC-MS/MS	Study of the differential expressed emmer seed proteins in response to fungal infection	Eggert et al. (2011)
In the spikes of six barley genotypes of varying resistance	3 d	Barley spikelets	2-DE, LC-MS/MS	Study of the differential expressed barley proteins in response to fungal infection	Geddes et al. (2008)
In the susceptible barley spikes grown under different N fertilizers	Maturity	Barley seeds	2-DE, MALDI MS/MS	Investigation of effect of nitrogen fertilizer mounts on the severity of FHB and identification of fungal proteins *in planta*	Yang et al. (2010a)
In the susceptible barley spikes	2 d	Barley spikelets	2-DE, MALDI MS/MS	Definition of infection levels correlated to fungal induced plant proteome degradation and identification of the differential expressed barley proteins in response to fungal infection	Yang et al. (2010b)
In the susceptible barley seeds	3 d	Germinating barley seeds	2-DE, MALDI MS/MS	Study of the differential expressed barley seed proteins in response to fungal infection during germination	Yang et al. (2011)
In the spikes of eleven barley genotypes of varying resistance	Maturity	Barley seeds	2-DE, LC-MS/MS	Study of the differential expressed barley seed proteins in response to fungal infection	Zantinge et al. (2010)
In the naked barley heads and co-colonization with *Fusarium culmorum*	Maturity	Naked barley grains	2-DE, MALDI MS, LC-MS/MS	Identification of the differentially expressed seed proteins in response to fungal infection and to growing location of the plant	Eggert and Pawelzik (2011)

Two phosphoproteome studies of *F. graminearum* under nitrogen limiting conditions and under conditions of unlimited nutrients have been published recently (Rampitsch et al., 2010, 2012). It was suggested that phosphorylation events are involved in the signaling pathways, leading to the activation of the trichothecene pathway, which is also activated in *F. graminearum* under nutrient stress (Rampitsch et al., 2010). A total of 348 phosphorylation sites localized to 301 peptides from 241 proteins including 10 protein kinases and seven transcription factors were identified during nitrogen starvation. When *F. graminearum* was grown *in vitro* without nutritional limitation, 2902 putative phosphopeptides with homologous matches to 1496 different proteins were identified (Rampitsch et al., 2012). Here, the majority of phosphoproteins were nuclear proteins with ATP-binding function and the phosphorylation sites were conserved in three phosphopeptides from transcription elongation factor 1β, acidic ribosomal proteins, and glycogen synthase.

Although it is very challenging to identify large numbers of *F. graminearum* proteins *in planta*, Paper et al. (2007) extracted *F. graminearum* secreted proteins from infected wheat heads by vacuum filtration, resulting in the identification of 120 fungal proteins including several cell wall degrading enzymes, of which 56% contained putative secretion signals. Additionally, proteomics analyses of *F. graminearum-*infected barley spikelets at maturity (Yang et al., 2010a) and 2 days after inoculation (dai; Yang et al., 2010b) as well as wheat spikelets from 1 to 3 dai (Zhou et al., 2006), revealed nine, one, and eight fungal proteins, respectively. The identification of fungal stress-related and antioxidant proteins *in planta* strongly suggests that the pathogen is exposed to stresses such as oxidation and starvation and that it attempts to overcome plant defense.

## Proteomics Studies of Host Defense to *Fusarium graminearum*

Extensive proteomics studies have been conducted in *F. graminearum*-infected wheat, barley, and their wild relatives (Table 1). With the exception of one recent study (Gunnaiah et al., 2012) using shotgun proteomics, other studies have employed gel-based techniques to investigate the differentially expressed proteins of hosts with different levels of disease susceptibility at different time points after inoculation at anthesis or during germination (Table 1). Due to the use of different cultivars, inoculation methods, infection stages, growth conditions, and proteomic techniques, little overlap is apparent between the regulated proteins identified in these studies. In resistant and/or susceptible wheat in response to *F. graminearum* up to 5 dai, many proteins related to carbon metabolism and photosynthesis were down-regulated, whereas the up-regulated proteins could be involved in antioxidant, jasmonic acid, and ethylene signaling pathways, phenylpropanoid biosynthesis, antimicrobial compound synthesis, detoxification, cell wall fortification, defense-related responses, amino acid synthesis, and nitrogen metabolism. Wheat susceptibility likely reflected the delayed activation of the salicylic acid defense pathway (Ding et al., 2011). Moreover, distinct abundance patterns of different xylanase inhibitor forms and pathogenesis-related (PR) proteins were shown in the wheat ear in response to the *F. graminearum* Δ*Tri5* mutant at 5, 15, and 25 dai (Dornez et al., 2010).

When the proteomes of mature grains of susceptible barley infected by *F. graminearum* under two different levels of nitrogen fertilizers were analyzed, massive, fungus-induced degradation of the grain proteome was observed and increased *Fusarium* infection occurred with low N amount (Yang et al., 2010a). In contrast, Zantinge et al. (2010) observed no degradation of seed proteomes of barley cultivars with different susceptibility to *F. graminearum*. These findings led to the analysis of the compatible interaction between barley and *F. graminearum* during the early infection stage to clearly define the infection levels correlated to the degree of fungal induced proteome degradation. Analysis of infected susceptible barley spikelets at 2 dai (Yang et al., 2010b) and 3 dai (Geddes et al., 2008) revealed up-regulation of proteins associated with oxidative stress response, PR-proteins and increased energy metabolism, although slight proteome degradation was observed at 2 dai (Yang et al., 2010b). However, changes in proteins associated with an oxidative response could not be observed in resistant barley (Geddes et al., 2008), suggesting enhanced oxidative stress in the compatible interaction. Moreover, investigation of infection of emmer wheat and naked barley mature grains by both *F. graminearum* and *F. culmorum* showed DON accumulation and several changed proteins involved in transcription regulation, defense responses, nutrient reservoirs, and starch biosynthesis in contrast to proteome degradation (Eggert and Pawelzik, 2011; Eggert et al., 2011). These results indicate that wild relatives can stimulate defense strategies in response to *Fusarium* infection after a long infection period up to the maturity stage.

## Contribution of Proteomics to Crop Protection Against FHB

In order to reduce epidemics of FHB, several approaches for control can be employed, including the use of cultural control techniques (e.g., crop rotations, irrigation, weed control, nitrogen input, and tilling), the use of fungicides, chemicals and biological control, transgenic plants, and resistance breeding (Parry et al., 1995). The major contribution of proteomics to crop protection is the identification of both fungal effectors possibly facilitating infection or triggering plant defense and host proteins or biomarkers possibly conferring enhanced resistance, which require subsequent functional analysis of corresponding genes to establish new strategies for disease control. So far, several *F. graminearum* genes relative to mycotoxin production, signal transduction, metabolism, and growth have been analyzed in detail to examine their roles in the virulence and pathogenicity (reviewed by Kazan et al., 2012), but the targets discovered on the basis of the outcomes of proteomics, which may be essential for fungal infection, have not been well investigated.

With respect to host resistance, proteomics has identified many host proteins in response to *F. graminearum*, the majority of which are often involved in primary metabolism, defense, and stress-related responses. However, the most frequently identified host proteins have not been fully investigated, except the PR-proteins (e.g., chitinase, β-1,3-glucanase and thaumatin-like protein), in terms of downstream characterization of their functional roles in enhanced resistance. The reasons can be that some proteins of interest are actually not found by proteomics due to low abundance or condition-dependent expression and that high-throughput stable transformation of wheat and barley for functional analysis of host genes is still not available. Furthermore, there has been a lack of wheat and barley genome sequences, although barley genome has recently been published (The International Barley Genome Sequencing Consortium, 2012), which should assist the process of identifying elements relating to resistance. Transgenic wheat expressing a α-1-purothionin, a thaumatin-like protein 1, a β-1,3-glucanase (MacKintosh et al., 2007), a class II chitinase (Shin et al., 2008), an antifungal plant defensin (Li et al., 2011), a pectin methyl-esterase inhibitor (Volpi et al., 2011), a polygalacturonase-inhibiting protein (Ferrari et al., 2012), a lactoferrin (Han et al., 2012), a *Arabidopsis thaliana* NPR1 (Makandar et al., 2006), or a truncated form of the yeast ribosomal protein L3 (Di et al., 2010) enhanced resistance to FHB under greenhouse conditions. However, transgenic lines have rarely been tested for FHB severity under field conditions. Among tested lines, a β-1,3-glucanase and a L3 transgenic wheat line conferred enhanced resistance in addition to reduced DON level in the L3 line. Transgenic barley expressing a FsTri101, a PDR5, a chitinase, or a thaumatin-like protein showed that neither of these genes was effective in the field at reducing FHB or DON levels, whereas two transgenic lines expressing another thaumatin-like protein or a trichothecene transporter, have shown reduced DON accumulation during 5 years of field trials (Dahleen et al., 2011). FHB resistance breeding is another approach, where mapping of QTLs controlling resistance to *F. graminearum* is a major task. Resistance-associated host metabolite-based breeding selection has also been suggested, when reliable associations between these metabolites as biomarkers and host resistance can be established (Bollina et al., 2011). The same principle can be applied to genes and proteins. Large-scaled “omics” studies of host-*F. graminearum* interactions offer such opportunity to identify potential biomarkers.

## Perspectives

Proteomics has become an indispensable tool for understanding molecular and cellular mechanisms in plant-microbe interactions. With the aid of the remarkable development of proteomics techniques, host genome sequencing and bioinformatics tools, the capability of proteomics to identify the novel elements involved in *F. graminearum* pathogenicity and virulence and host resistance will continue to improve. However, the full characterization of a proteome is extremely challenging due to proteome dynamics and complexity. The high cost and complexity of experimental procedures also limit the utilization of proteomics. Furthermore, functional analysis of identified proteins or genes is required to elucidate their roles in pathogenicity and plant resistance. Although significant progress has been made in understanding FHB, environmental changes, and evolution of virulence and toxin biosynthesis in *F. graminearum* are highly challenging for disease control. Therefore, it will be essential to integrate all the information generated from the “omics” studies together with plant pathology and genetic engineering to fully understand host *F. graminearum* interactions for development of sustainable cereal protection strategies.

## Conflict of Interest Statement

The authors declare that the research was conducted in the absence of any commercial or financial relationships that could be construed as a potential conflict of interest.
